# A robust approach to optimizing multi-source information for enhancing genomics retrieval performance

**DOI:** 10.1186/1471-2105-12-S5-S6

**Published:** 2011-07-27

**Authors:** Qinmin Hu, Jimmy Xiangji Huang, Jun Miao

**Affiliations:** 1 Information Retrieval and Knowledge Management Research Lab, York University, Toronto, ON, M3J1P3, Canada; 2 Department of Computer Science & Engineering, York University, Toronto, ON, M3J1P3, Canada; 3 School of Information Technology, York University, Toronto, ON, M3J1P3, Canada

## Abstract

**Background:**

The users desire to be provided short, specific answers to questions and put them in context by linking original sources from the biomedical literature. Through the use of information retrieval technologies, information systems retrieve information to index data based on all kinds of pre-defined searching techniques/functions such that various ranking strategies are designed depending on different sources. In this paper, we propose a robust approach to optimizing multi-source information for improving genomics retrieval performance.

**Results:**

In the proposed approach, we first consider a common scenario for a metasearch system that has access to multiple baselines with retrieving and ranking documents/passages by their own models. Then, given selected baselines from multiple sources, we investigate three modified fusion methods in the proposed approach, reciprocal, CombMNZ and CombSUM, to re-rank the candidates as the outputs for evaluation. Our empirical study on both 2007 and 2006 genomics data sets demonstrates the viability of the proposed approach for obtaining better performance. Furthermore, the experimental results show that the reciprocal method provides notable improvements on the individual baseline, especially on the passage2-level MAP and the aspect-level MAP.

**Conclusions:**

From the extensive experiments on two TREC genomics data sets, we draw the following conclusions. For the three fusion methods proposed in the robust approach, the reciprocal method outperforms the CombMNZ and CombSUM methods obviously, and CombSUM works well on the passage2-level when compared with CombMNZ. Based on the multiple sources of DFR, BM25 and language model, we can observe that the alliance of giants achieves the best result. Meanwhile, under the same combination, the better the baseline performance is, the more contribution the baseline provides. These conclusions are very useful to direct the fusion work in the field of biomedical information retrieval.

## Background

Through the use of information retrieval (IR) technologies, information systems retrieve information to index data based on all kinds of pre-defined searching techniques/functions. Each information system has its own models to rank the output. A metasearch system will get access to multiple IR systems and combine their ranking results into a single ranking output generated by the metasearch system. Metasearch systems do not crawl the raw data or maintain a database as most IR systems do, but instead they search several IR systems simultaneously, which act as an agent to pass the query to the search systems and then return the results. Since there are different results retrieved by IR systems/models, metasearch systems provide a quick way to determine which systems are retrieving the best match for information needs. The major goal of the TREC Genomics Tracks is to create test collections for evaluation of IR and its related tasks in the genomics domain. The users desire to be provided short, specific answers to questions and put them in context by providing linking to original sources from the biomedical literature. This motivates the TREC Genomics Track to implement a new task in 2006 that focuses on passage retrieval using full-text documents from the biomedical literature [1]. For the TREC 2006 and 2007 Genomics Track, systems are tasked with extracting out relevant passages of text that answer topic questions and focus on retrieval of short passages (from phrase to sentence to paragraph in length) that specifically address an information need, along with linkage to the location in the original source document [1,2]. Here a passage is defined to be a string of characters within a natural paragraph [1]. Systems are not only tasked to return passages of text, but also measured on how well they retrieve relevant information at the document-level, aspect-level and passage2-level, which will be presented in the results and discussion section.

In the TREC 2007 Genomics Track, there are a total of 66 runs submitted, in which 49 are classified as automatic. Among the 49 submitted runs, submissions have employed multiple approaches for retrieval processes, such as query expansion, various levels of passage retrieval granularity, and varying IR models with many different scoring schemes. Therefore, meta-features are distilled from the submissions as high-level categories, which are shown in Table 1[2]. For example, “TfidfIR” uses passage retrieval by a vector space model with any variant of TF-IDF [3], “OkapiIR” indicates passage retrieval using an Okapi BM25 model [4,5], “LmIR” means passage retrieval using a language model, and “FusionIR” combines results from two or more systems regardless of fusion operator usage. This motives us to consider a multi-source fusion approach in a metasearch system to utilize these meta-features. In addition, The performance of NLMFusion, the top scoring automatic run for all three measures (the document-level, the passage2 level and the aspect-level) in 2007 [2], suggests that combining results from different IR models may improve the final results [2].

**Table 1:** Meta-Features of Runs

Meta-Feature	Description
FusionIR	fusion - combining results from 2 or more systems regardless of fusion operator used
OkapiIR	passage retrieval using an Okapi BM25 model
TfIdfIR	passage retrieval using a vector space model with any variant of TF-IDF
LmIR	passage retrieval using any language model
DfrIR	passage retrieval using a vector space model with any variant of divergence from randomness (DFR)
...	...

In the TREC 2007 Genomics Track, submissions have employed multiple approaches for retrieval processes, such as query expansion, various levels of passage retrieval granularity, and varying IR models with many different scoring schemes. This table presents five typical and popular meta-features as follows.

In this paper, we propose a robust approach to combining multiple IR baselines from multiple sources in the genomics domain. First, the proposed approach employs three modified fusion methods, reciprocal, CombMNZ and CombSUM, where CombMNZ is generated into three versions to deeply evaluate this popular combination method. Second, considering the diversity of baselines, we assume the proposed approach in the metasearch system has access to the baselines from three kind of individual models, DFR, BM25 and language model. Therefore, we select five baselines from the official submissions of the TREC 2007 Genomics Track for combination as the main part of our experiments. Third, in order to evaluate the superiority of the proposed approach, we conduct the experiments not only on the base runs from different sources, but also on the baselines from a single source of Okapi BM25 with different indices, using the 2007 and 2006 genomics data sets. Fourth, the experimental results demonstrate the viability and superiority of the propose approach with reciprocal to better performance fusion. In addition, as an extension of our preliminary work [6], we employ CombSUM as the third combination method and further evaluate CombMNZ by considering its normalization, assigned weights and multiple times application.

The paper is organized as follows. First, we briefly present the experimental results and discussions in the results and discussion section, where the IR environment is introduced with the descriptions of the data sets, queries and evaluation measures. The comprehensive empirical study includes the analysis for the baselines, the proposed approach, the comparisons of CombMNZ and CombSUM to reciprocal, and the influence of the proposed approach on the single model BM25. Second, we show our contributions in the conclusion section. Third, in the methods section, we propose our methods systematically and consistently. A robust approach to optimizing multi-source IR systems is proposed, followed by the introductions of reciprocal, CombMNZ and CombSUM, the descriptions of IR models as DFR, BM25 and language model. The related work is also presented in this section.

## Results and discussion

In this section, we conduct a series of pilot experiments using reciprocal, CombMNZ and CombSUM on the 2007 and 2006 genomics data sets.

### IR environment

#### Data sets and queries

We evaluated our model and algorithms on the 2007 and 2006 TREC data sets. The TREC 2007 and 2006 Genomics data sets provide a test collection of 162,259 full-text documents assembled with 36 queries in 2007 and 28 queries in 2006. The TREC 2007 queries are in the form of questions asking for lists of specific entities. The definitions for these entity types are based on controlled terminologies from different sources, with the source of the terms depending on the entity type [2]. The TREC 2006 queries are derived from the set of biologically relevant questions based on the Generic Topic Types (GTTs) [7]. There is a sample query as Query 200 as “What serum [PROTEINS] change expression in association with high disease activity in lupus?”. More information is available on the official genomics website at: http://ir.ohsu.edu/genomics.

#### Evaluation measures

The TREC Genomics Track has three evaluation measures that are the document-level, the aspect-level and the passage2-level (a new measure for the TREC 2007 queries) [2]. Each of these provides insight into the overall performance for a user trying to answer the given queries and measured by some variant of mean average precision (MAP), which are briefly described as follows.

##### Document-level

This is a standard IR measure. The precision is measured at every point where a relevant document is obtained and then averaged over all relevant documents to obtain the average precision for a given query. For a set of queries, the mean of the average precision for all queries is the mean average passage precision of that IR system.

##### Aspect-level

A question could be addressed from different aspects. For example, the question “what is the role of gene PRNP in the Mad cow disease?” could be answered from aspects like “Diagnosis”, “Neurologic manifestations”, or “Prions/Genetics”. This measure indicates how comprehensive the question is answered [1].

##### Passage2-level

This is a new character-based MAP measure which is added to compare the accuracy of the extracted answers and modified from the original measure Passage MAP. Passage2 treats each individually retrieved character in published order as relevant or not, in a sort of “every character is a mini relevance-judged document” approach [2]. This is done to increase the stability of the passage MAP measure against arbitrary passage splitting techniques.

### Performance of official baselines

Table 2 presents the performance of five selected baselines which are the official submissions in the TREC 2007 Genomics Track. The models applied in each baseline are specified in the parentheses as “DFR”, “BM25” and “LM”. Here “LM” stands for “language model”. We can see that “MuMshFd” and “UBexp1” have better performance than “york07ga2” and “kyoto1”. We choose these baselines in a performance range in order to check what kind of combination will be most effective. More details will be discussed in the following sections.

**Table 2:** Baseline Performance

baseline	document	aspect	passage2
UniNE1 (DFR)	0.2777	**0.2189**	**0.0988**
MuMshFd (BM25)	**0.2906**	0.2068	0.0895
york07ga2 (BM25)	0.2150	0.1306	0.0472
kyoto1 (LM)	0.1892	0.1208	0.0209

The performance of five selected baselines is presented in the following table. The baselines are the official submissions in the TREC 2007 Genomics Track. The model applied in each baseline is specified in the parentheses as “DFR”, “BM25” and “LM”. Here “LM” stands for “language model”.

### Influence of reciprocal

Corresponding to the baselines, we evaluate the combinations applying the reciprocal method. Due to three kind of IR models, there are four combinations as listed in Table 3. Each combination contains a DFR baseline, a BM25 baseline and a LM baseline. The values in the parentheses are the relative rates of improvement over the best results of the baselines.

**Table 3:** Reciprocal Performance

Component	document	aspect	passage2
Best of baselines	0.2906	0.2189	0.0988

UniNE1+York07ga2+kyoto1	0.2743 (-5.60%)	0.2065 (-5.63%)	0.0978 (-1.01%)
UniNE1+York07ga2+UBexp1	0.2802 (-3.56%)	0.2219 (1.38%)	0.1047 (5.96%)
UniNE1+MuMshFd+kyoto1	0.2828 (-2.66%)	0.2221 (1.46%)	0.0997 (0.86%)
**UniNE1+MuMshFd+UBexp1**	**0.2906** (0.00%)	**0.2380 (8.75%)**	**0.1059 (7.19%)**

Corresponding to the baselines, we evaluate the combinations using the reciprocal method in this table. In total, there are four combinations generated from three different IR models. Each combination contains a DFR baseline, a BM25 baseline and a LM baseline. The values in the parentheses are the relative rates of improvement over the best results of the baselines. One of the conclusions is that the alliance of giants with boldface is the winner on all the measures.

First, the reciprocal method works very well on the passage2-level and the aspect-level, while it does not contribute a lot on the document-level. Second, “UniNE1+MuMshFd+UBexp1” achieves the best performance, especially in terms of the passage2-level. As we note in Table 2, “MuMshFd” and “UBexp1” have better performance than “york07ga2” and “kyoto1”. We can see that the alliance of giants is the winner on all the measures. In addition, for the overall performance on the passage2-level, the performance generated by the alliance of giants “UniNE1+MuMshFd+UBexp1”, almost catches up with the top official automatic run, “NLMfusion” [8]. Note that “NLMFusion” is an automatic run obtained by five baselines, instead of three in our experiments.

In Table 3, both “UniNE1+MuMshFd+UBexp1” and “UniNE1+York07ga2+UBexp1” make improvements in terms of the passage2-level and the aspect-level. Focusing on the passage2-level, we can see that the different components of these two combinations are the BM25 baselines, “york07ga2” and “MuMshFd”. Then we can argue that the language model “UBexp1” contributes more than the BM25 model “MuMshFd” in the proposed approach. This conclusion can also be confirmed by comparing “UniNE1+York07ga2+UBexp1” with “UniNE1+MuMshFd+kyoto1”, in which the latter one has better performance than the preceding one.

Furthermore, a common conclusion can also be drawn that the baselines who have better performance effect the combination results more significantly. For example, the alliance of giants “UniNE1+MuMshFd+UBexp1”, which has the best DFR run, the best BM25 run and the best language model run, achieves the best fusion result. “UniNE1+MuMshFd+kyoto1” is better than “UniNE1+york07ga2+kyoto1”, because “MuMshFd” is better than “york07ga2”.

### Comparison to combMNZ

Table 4 presents the performance of applying the CombMNZ method. In order to deeply evaluate the benefits of CombMNZ, we introduce three versions as CombMNZ-with-normalization, CombMNZ-with-assigned-weight and CombMNZ-with-multiple respectively. The values in the parentheses are the relative rates of improvement over the best results of the baselines.

**Table 4:** Performance of CombMNZ

Components	w/ Normalization			w/ Assigned Weights			w/ Multiple		
	document	aspect	passage2	document	aspect	passage2	document	aspect	passage2
Best of baselines	0.2906	0.2189	0.0988	0.2906	0.2189	0.0988	0.2906	0.2189	0.0988

UniNE1+York07ga2+kyoto1	0.2671 (-8.08%)	0.1535 (-29.86%)	0.0937 (-5.13%)	0.2729 (-6.09%)	0.1854 (-15.27%)	0.0957 (-3.19%)	0.2571 (-11.53%)	0.1547 ( -29.33% )	0.0924 ( -6.49%)
UniNE1+York07ga2+UBexp1	0.2656 (-8.61%)	0.1772 (-19.03%)	0.0879 (-10.99%)	0.2591 (-10.82%)	0.1878 (-14.18%)	0.0867 (-12.30%)	0.2639 (-9.16%)	0.1753 (-19.92%)	0.0885 (-10.43%)
UniNE1+MuMshFd+kyoto1	0.2559 (-11.95%)	0.1801 (-17.70%)	0.0985 (-0.30%)	0.2503 (-13.85%)	0.1837 (-16.09%)	0.0908 (-8.06%)	0.2401 (-17.38%)	0.1599 (-26.96%)	0.0958 (-3.04%)
UniNE1+MuMshFd+UBexp1	0.2416 (-16.85%)	0.1720 (-21.43%)	0.0871 (-11.86%)	0.2466 (-15.11%)	0.1787 (-18.36%)	0.0839 (-15.09%)	0.2419 (-16.74% )	0.1716 (-21.61% )	0.0872 (-11.72%)

In order to deeply evaluate the benefits of CombMNZ, we generate CombMNZ-with-normalization, CombMNZ-with-assigned-weight and CombMNZ-with-multiple respectively. For CombMNZ-with-normalization, we employ the standard zero-one normalization method in which all the base weights are scaled between zero being the lowest value and one being the absolute highest value. For CombMNZ-with-assigned-weight, the baselines earn their weights depending on their models. Only the optimal results are presented. For CombMNZ-with-multiple, we apply the CombMNZ method for multiple times (*m* times, where *m* is set to be one of {1, 2, 3, 5}). No normalization and no additional weights has been given to the baselines. Only the optimal results are presented as well. The values in the parentheses are the relative rates of improvement over the best results of the baselines. Note that “w/” stands for “with”.

In CombMNZ-with-normalization, we employ the standard zero-one normalization method in which all base weights are scaled between zero being the lowest value and one being the absolute highest value. CombMNZ-with-normalization is the most popular version such that we generate another two versions of CombMNZ to check its effectiveness.

In CombMNZ-with-assigned-weight, the baselines earn their weights depending on their models. For *N* baselines, different weights are assigned to them linearly, in which the sum of the weights equals to one always. In this paper, we conduct the experiments with tuning the assigned weights. Only the optimal results are presented in Table 4.

In CombMNZ-with-multiple, we apply the CombMNZ method for multiple times. In the experiments, we try *m* times (where *m* is set to be one of {1, 2, 3, 5}) on the baselines. No normalization and additional weights has been given to the baselines. Only the optimal results are presented in Table 4 as well.

Although CombMNZ has been confirmed by Lee [9], Fox and Shaw [10] as an effective method. However, in our experiments in the biomedicine domain, CombMNZ does not show any advantage at all, although three different versions have been generated. In Table 4, all the combinations get worse compared with the best results of the baselines, especially in terms of the passage2-level and the aspect-level. On the genomics data, reciprocal outperforms CombMNZ thoroughly.

### Comparison to combSUM

Fox and Shaw [10] proved that the CombSUM method can achieve good performance on the TREC-2 data set. In this paper, we apply CombSUM as a second comparison to reciprocal, since CombMNZ doesn’t work on the genomics data set.

In Table 5, CombSUM does not work very well on the baselines. However, the alliance of giants “UniNE1+MuMshFd+UBexp1” outperforms the best baseline on the passage2-level. We can say that the CombSUM method has great potential to improve the retrieval performance on multi-source baselines in the genomics domain. Compared to reciprocal, reciprocal outperforms CombSUM on all the measures as well. Although both CombSUM and CombMNZ do not work as well as reciprocal, CombSUM provides its effectiveness better than CombMNZ with the evidence of the improved passage2-level performance.

**Table 5:** Performance of CombSUM

Component	document	aspect	passage2
Best of baselines	0.2906	0.2189	0.0988

UniNE1+York07ga2+kyoto1	0.2692 (-7.36%)	0.1552 (-29.07%)	0.0939 (-4.94%)
UniNE1+York07ga2+UBexp1	0.2690 (-7.41%)	0.1840 (-15.94%)	0.0944 (-4.49%)
UniNE1+MuMshFd+kyoto1	0.2567 (-11.66%)	0.1809 (-17.35%)	0.0985 (-0.30%)
UniNE1+MuMshFd+UBexp1	0.2630 (-9.49%)	0.1919 (-12.32%)	0.0991 (0.30%)

We evaluate the combinations applying the CombSUM method in this table. The values in the parentheses are the relative rates of improvement over the best results of the baselines.

Furthermore, the application of CombSUM repeatedly confirms that the alliance of giants achieves the best results over the other combinations. In addition, comparing “UniNE1+MuMshFd+kyoto1” with “UniNE1+MuMshFd+UBexp1”, we can see the evidences as no big performance gap on all the measures and only a different component between them. Then a conclusion can be drawn that “UBexp1” doesn’t contribute much more than “kyoto1”, although “UBexp1” outperforms “kyoto1” much. On the other hand, comparing “UniNE1+York07ga2+UBexp1” with “UniNE1+MuMshFd+UBexp1”, we also get the evidences as big performance gap existing especially on the passage2-level and only a different component between them. Then another conclusion can be drawn that “MushMshFd” contributes much more than “York07ga2”, since “MushMshFd” has much better performance than “York07ga2”.

### Influence of the proposed approach on the single source

In the previous sections, we evaluate our proposed approach on the official multi-source submissions of the REC 2007 Genomics track. Among three different models, the reciprocal method obtains nice performance as a good combination method. In this section, we will examine how our proposed approach works based on the single source of Okapi BM25.

First of all, the baselines are from three different indices under the same IR model, BM25, instead of those from three kind of IR models. Second, three indices are built on the 2007 and 2006 genomics data sets according to three passage extraction methods [11-13]. Here “word” stands for “word-base”, “sentence” for “sentence-base” and “paragraph” for “paragraph-base”. Third, the Okapi tuning parameters of the selected runs are (*k*_1_, *b*) = (0*.*5, 1*.*3). Similarly, reciprocal, CombMNZ and CombSUM are applied as the same way in the previous experiments. Table 6 shows the performance of baselines and combinations in 2007 and 2006 respectively.

**Table 6:** Performance of the Fusion Approach on Okapi 2007 and 2006

Components	Okapi 2007			Okapi 2006	
	document	aspect	passage2	document	aspect
word	0.2108	0.1080	0.0364	0.3140	0.1237
sentence	0.1805	0.0970	0.0350	0.3030	0.1206
paragraph	0.1588	0.0616	0.0333	0.3109	0.1410

reciprocal	**0.2219 (5.29%)**	**0.1237 (14.51%)**	**0.0478 (31.40%)**	**0.3168 (1.07%)**	**0.1449 (12.25%)**
CombMNZ-with-normalization	0.1703 (-19.20%)	0.0643 (-40.43%)	0.0270 (-25.92%)	0.2352 (-26.55%)	0.0498 (-61.46%)
CombMNZ-with-assigned-weights	0.1777 (-15.72%)	0.0701 (-35.12%)	0.0273 (-24.88%)	0.2441 (-23.78%)	0.0524 (-59.43%)
CombMNZ-with-multiple	0.1730 (-17.93%)	0.0651 (-39.73%)	0.0277 (-24.01%)	0.2375 (-25.85%)	0.0508 (-60.62%)
CombSUM	0.1818 (-13.76%)	0.0718 (-33.56%)	0.0297 (-18.43%)	0.2559 (-20.10%)	0.0719 (-44.32%)

We examine the proposed robust approach on the single model with Okapi BM25. First of all, the baselines are from three different indices under the same IR model, BM25, instead of those from three kind of IR models. Second, three indices are built on the 2007 and 2006 genomics data sets according to three passage extraction methods [11,12]. Here “word” stands for “word-base”, “sentence” for “sentence-base” and “paragraph” for “paragraph-base”. Third, the Okapi tuning parameters of the selected runs are (*k*_1_, *b*) = (0.5, 1.3). The values in the parentheses are the relative rates of improvement over the best results of the baselines.

In the TREC 2007 Genomics Track overview [14], the measure correlation of the four measures shows that the passage2-level is highly correlated with the aspect-level. Therefore, on the 2006 data set, we choose the aspect-level as our main measure, since there is no passag2-level in 2006. Focusing on the passage2-level and the aspect-level, we can observe the reciprocal method outperforms CombMNZ and CombSUM obviously in Table 6. The reciprocal method achieves great improvements on the passage2-level, the aspect-level and the document-level on both 2007 and 2006 genomics data sets. The standard normalization method, tuning the assigned weights and using multiple times CombMNZ can not help CombMNZ to make progress on the 2007 and 2006 data sets respectively. CombSUM does not work well on both 2007 and 2006 data sets. However, the consistent conclusion can be drawn that the CombSUM method works slightly well than the CombMNZ method, although both of them are not as good as reciprocal.

## Conclusions

In this paper, we propose a robust approach with multi-source information for improving IR performance in the genomics domain. The proposed approach employs a reciprocal method, a CombMNZ method and a CombSUM method respectively, with evaluation on the TREC 2007 and 2006 genomics data sets. Empirical study on three different IR models demonstrates the utility of our proposed approach.

Compared to the CombMNZ and CombSUM methods, the reciprocal method provides notable improvements using the baselines from a DFR model, a BM25 model and a language model respectively. The improvements are significant for both TREC 2007 and 2006 genomics data set, in which the improved result in terms of the passage2-level in 2007 almost catches up with the highest official result “NLMFusion” [8]. While CombMNZ does not achieve good performance, we conduct three versions as CombMNZ-with-normalization, CombMNZ-with-assigned-weight and CombMNZ-with-multiple to further improve and evaluate the CombMNZ method. Although the CombSUM method does not work as well as reciprocal, CombSUM makes progress on the passage2-level, also works better than CombMNZ on all the three versions.

We select five baselines from three kind of IR models as DFR, BM25 and language model. The experimental results implement the following conclusions: 1) the alliance of giants achieves the best result; 2) under the same combination, the better the baseline performance is, the more contribution the baseline provides.

Furthermore, the proposed robust approach makes improvements not only for combining the baselines from different sources, but also for combining the baselines from the single source such as Okapi BM25.

## Methods

In this section, we first define a baseline combination problem formally. Then, we introduce three modified methods of reciprocal, CombMNZ and CombSUM respectively. After that, we give a brief review for three IR models of DFR, BM25 and language model. Finally, we present the related work in details.

### Problem definition

In this paper we focus on exploring a multi-source fusion approach for a metasearch system, where the metasearch approach has access to multiple IR systems that retrieve and rank documents/passages with their own models. We are interested in a scenario in which the proposed approach only concerns the baselines retrieved by the IR models and then re-rank the results as the output for evaluation.

For simplicity, throughout this paper, we will assume that our proposed approach works on three kind of baselines: 1) a DFR baseline, *B*_1_; 2) a BM25 baseline, *B*_2_ and 3) a language model baseline, *B*_3_. Furthermore, we will select these baselines from the official submissions of the TREC 2007 Genomics Track. In addition, considering the performance range and effectiveness of the baselines, we try to choose more than a base run with the higher/lower performance. Since DFR is often used in fusion as one of the components, there is only a run named “UniNE1” from University of Neuchatel [15] which used DFR as a single model but did not combine many other models. Hence, we choose “UniNE1” as a seed *B*_1_ of DFR in the proposed metasearch system. For BM25, we choose two baselines as “MuMshFd”, *B*_21_ from University of Melbourne [16] and “york07ga2”, *B*_22_ from York University [17]. And we choose two language model baselines as “UBexp1”, *B*_31_ from University Buffalo [18] and “kyoto1”, *B*_32_ from Kyoto University [19]. Hence, given a query *q*, we put all retrieval documents by three baselines *B*_1_, *B*_2_*_i_* and *B*_3_*_j_* (where *i*, *j* = 1, 2) as *D*, the corresponding weights of the documents as *R*. Based on the combination methods, reciprocal, CombMNZ and CombSUM, the proposed approach re-ranks the documents/passages as the new output.

### Reciprocal

Our intuition in choosing the reciprocal method as the formula in Equation 1, derives from the fact of an exponential function, while highly ranked documents are more important than the lower ranked documents. Reciprocal simply sorts the documents according to a naive scoring formula. Given a set *D* of documents to be ranked and a set of rankings *R*, for each permutation on 1*..|D|*, we compute(1)

where *r*(*d*) stands for the weight of the document, and the constant *k* mitigates the impact of high weights. We also fixed *k* = 60 [20] during a pilot investigation and not altered during subsequent validation, which will not be discussed because of the limit space.

### CombMNZ

Fox and Shaw [10] introduced several combination methods such as CombMax, CombMin, CombSUM, CombANZ, CombMNX and CombMed, and they found CombSUM to be the best performing combination method. Lee [9] conducted extensive experiments with Fox and Shaw combination method based on the TREC data, and he found CombMNZ emerges as the best combination method. In this paper, we apply CombMNZ in the proposed approach as part of the proposed fusion framework.

CombMNZ requires for each *r* a corresponding scoring function *s_r_* : *D* → *R* and a cutoff rank *c* which all contribute to the CombMNZ score:(2)

### CombSUM

As one of the famous combination methods proposed by Fox and Shaw [10], CombSUM is defined as the summation of the set of similarity values, or, equivalently, the numerical mean of the set of the set of similarity values. In [10], the CombSUM method made the significant improvements over all the baselines such that CombSUM is claimed to perform better than the rest of other methods such as CombMIN, CombANZ on the TREC-2 data set. In the image retrieval domain, Chatzichristofis et al. [21] also proved that the CombSUM method was beneficial to improve image information retrieval performance. In this paper, we employ the CombSUM method to evaluate its effectiveness on the genomics domain.

### IR Systems

In this section, we give a brief review for three well-known weighting models as the Okapi BM25 [22], language model [23,24], and DFR [25].

#### Divergence from randomness

where *IG* is the information gain, which is given by a conditional probability of success of encountering a further token of a given word in a given document on the basis of the statistics on the retrieved set. *Prob*(*tf*) is the probability of observing the document *d* given *tf* occurrences of the query term *t. –log*_2_*Prob*(*tf*) measures the amount of information that term *t* carries in *d. qtw* is the query term weight component. Similarly to the query model in language modeling [24], *qtw* measures the importance of individual query terms. In the DFR framework, the query term weight is given by:(4)

where *qtf*(*t*) is the query term frequency of *t*, namely the number of occurrences of *t* in the query. *qtf_max_* is the maximum query term frequency in the query.

The other two components, namely information gain (IG) and information amount (*–log*_2_*Prob*(*tf*)), can be approximated by different statistics so that various instantiations of DFR are implemented.

#### Okapi BM25

where *w* is the weight of a query term, *N* is the number of indexed documents in the collection, *n* is the number of documents containing the term, *R* is the number of documents known to be relevant to a specific topic, *r* is the number of relevant documents containing the term, *tf* is within-document term frequency, *qtf* is within-query term frequency, *dl* is the length of the document, *avdl* is the average document length, *nq* is the number of query terms, the *k_i_*s are tuning constants (which depend on the database and possibly on the nature of the queries and are empirically determined), *K* equals to *k*_1_* ((1 *– b*) + b * dl/avdl)*.*

#### Language model

where *w* is the weight of a query term, *tf* is within-document term frequency, *FreqTotColl* is within-collection term frequency, *l* is document length, *F_t_* is length of the whole collection, the *mu* is tuning constants.

### Related work

A lot of previous work has been done on result combination. In the TREC 2007 Genomics Track, there are more than seven teams which utilize result combination to improve their final submissions in a total of 66 runs by 27 teams. “NLMFusion”, submitted by the team of National Library of Medicine [8], as the top scoring automatic run for all three metrics of the passage2-level, the aspect-level and the document-level, suggested that combining results from different IR models may improve the final score. Here “NLMFusion” is an automatic run obtained by applying fusion to a LHNCBC run, a Terrier run, an NCBI Themes run, an INDRI run and an easyIR run. However, not all teams using fusion/combination achieved the successfully improvements. The teams from University of Neuchatel [15], European Bioinformatics Institute [26], Kyoto University [19] and so on, showed slight declines in performance from their non-fusion/non-combination runs. Nevertheless, each team who used different methods, for fusing the individual different method runs, may have contributed to the differences in performance.

Divergence from randomness (DFR) [3], as one of five individual runs used in “NLMFusion”, was reported to be the highest scoring subcomponent run in the TREC 2007 Genomics Track. “UniNE3” [15], the fusion run submitted by University of Neuchatel, also gave details of success in using it. Since DFR was often used in fusion as one of the components, such as in 49 automatic submissions in 2007, there was only a run as “UniNE1” from University of Neuchatel [15] which used DFR as a single model but did not combine too many other models.

Okapi BM25, as one of the best well-known probabilistic weighting function, was very popular in the TREC Genomics Tracks. “MuMshFd”, the run submitted by University of Melbourne [16], obtained the highest score of the passage2-level, the aspect-level and the document-level in all the BM25 submissions. Other teams who applied the Okapi BM25 model, such as those from York University [17] and University of Illinois at Chicago [27], obtained the performance around the mean MAP on all the evaluation measures. “DUTgen3”, submitted by Dalian University of Technology [28], which also used the Okapi BM25 model, however, only slightly hit the median MAP.

Language model, as one of the most well-known statistical model, was also employed popularly by many teams. “AIDrun3” submitted by Arizona State University [14], “DUTgen1” and “DUTgen2” submitted by Dalian University of Technology [28], “UBexp1” from University at Buffalo [18] and “kyoto1” from Kyoto University [19], achieved better average performance than the Okapi runs, although the individual run is not as good as the Okapi BM25 run, “MuMshFd” submitted by University of Melbourne.

## Competing interests

The authors declare that they have no competing interests.

## Authors' contributions

This is a featuring work done by QH as a part of her Ph.D. thesis. JXH supervised the project and revised the manuscript. JXH and JM contribute in the study design and experiments. All authors read and approved the final manuscript.
